# Automatic Reading of ANCA-Slides: Evaluation of the AKLIDES System

**DOI:** 10.1155/2012/762874

**Published:** 2012-11-05

**Authors:** Jan Damoiseaux, Kathleen Mallet, Mia Vaessen, Jos Austen, Jan Willem Cohen Tervaert

**Affiliations:** Laboratory of Clinical Immunology, Maastricht University Medical Center, P. Debyelaan 25, 6229 HX Maastricht, The Netherlands

## Abstract

The ANCA consensus prescribes screening by indirect immunofluorescence on neutrophils. We evaluated the first automated ANCA-pattern recognition system. C-ANCA (*n* = 39) and P-ANCA (*n* = 40) samples were selected from patients with ANCA-associated vasculitis (AAV). Non-AAV controls included sera from healthy controls (*n* = 40), sera with possible interfering antibodies (*n* = 46), or miscellaneous ANCA reactivity (*n* = 31). ANCA slides were analysed by AKLIDES and routine fluorescence microscopy. The C-ANCA pattern was recognized by routine microscopy in 92% and 97% on ethanol- and formalin-fixed slides, respectively. AKLIDES reported C-ANCA in 74% and 95%, respectively. P-ANCA was recognized by routine microscopy on ethanol-fixed neutrophils in 90%, while AKLIDES reported P-ANCA in 80%. Typically, only 65% and 33% of these samples showed the expected C-ANCA on formalin-fixed neutrophils by routine microscopy and AKLIDES, respectively. A C- or P-ANCA pattern was observed on ethanol-fixed neutrophils in 28% and 23% of the controls by routine microscopy and AKLIDES, respectively. Only 5% of the controls revealed C-ANCA on formalin-fixed neutrophils by routine microscopy and AKLIDES. Altogether, automated ANCA-pattern recognition by AKLIDES is promising. Distinction of C- and P-ANCA is good, but sensitivity on ethanol-fixed neutrophils needs improvement. When optimized, pattern recognition software may play an important role in AAV diagnostics.

## 1. Introduction

Detection of antineutrophil cytoplasmic antibodies (ANCAs) is relevant for the diagnosis of the ANCA-associated vasculitides (AAV), including granulomatosis with polyangiitis (GPA, previously referred to as Wegener's granulomatosis), eosinophilic granulomatosis with polyangiitis (EGPA; previously referred to as the Churg-Straus syndrome), microscopic polyangiitis (MPA), and renal-limited necrotizing crescentic glomerulonephritis (NCGN) [1]. Classification criteria for these diseases have been defined by the American college of rheumatology (ACR) [2] and the Chapel Hill consensus conference [3]. The presence of ANCA, however, is not part of these criteria which are primarily based on clinical manifestations and histopathology as observed in biopsies obtained from the affected tissues. More recently, a novel consensus methodology for the classification of AAV was developed and validated for epidemiological studies [4]. Importantly, the latter classification criteria incorporated the ANCA status of the patient.

The current international consensus on ANCA testing prescribes screening by indirect immunofluorescence (IIF) on ethanol-fixed neutrophils [5]. Four different patterns can be distinguished. First, the classical (C-)ANCA is characterized by a granular, cytoplasmic fluorescence with central or interlobular accentuation; second, a diffuse flat cytoplasmic fluorescence without interlobular accentuation may be referred to as atypical C-ANCA. In clinical practice, however, both patterns are difficult to distinguish and many clinical laboratories do label both these patterns as “C-ANCA.” Third, the perinuclear (P-)ANCA is characterized by perinuclear staining, with or without nuclear extension. Reading of the P-ANCA pattern may be hampered by the presence of interfering antinuclear antibodies (ANAs). The perinuclear staining pattern actually is an artefact, since formalin-fixation results in a cytoplasmic staining pattern, indistinguishable from C-ANCA on ethanol-fixed neutrophils. Finally, if a combination of cytoplasmic and perinuclear staining occurs, this is called atypical ANCA. Importantly, in AAV it is mandatory to establish with antigen-specific assays that ANCAs are directed either to serine protease 3 (PR3) or myeloperoxidase (MPO) for optimal diagnostic performance [1, 5, 6].

IIF is a labour-intensive technique, requires special expertise of the technician, and is hampered by the subjective reading of the slides [7]. The advent of microscope devices with integrated software for pattern recognition might overcome this problem [8]. The AKLIDES system is the first automated system for ANCA-pattern recognition based on the combination of ethanol- and formalin-fixed ANCA slides. In this study, we have evaluated the AKLIDES system using sera from AAV patients (*n* = 79) as well as distinct cohorts of relevant control sera (*n* = 117).

## 2. Materials and Methods

### 2.1. Patient Sera

Samples of AAV patients were selected based on the routine ANCA IIF analysis using ethanol-fixed ANCA slides (INOVA, San Diego, CA, USA) [9]. Samples with a C-ANCA pattern (*n* = 39) were selected from AAV patients (25 males and 14 females, median age 58 yrs, range 20–83 yrs) that were PR3-ANCA-positive at the time of diagnosis; titres varied from 1/32 up to >1/1024. Similarly, samples with a P-ANCA pattern (*n* = 40) were selected from AAV patients (25 males and 15 females, median age 60 yrs, range 19–78 yrs) that were MPO-ANCA-positive at the time of diagnosis; titres varied from 1/32 up to ≥1/1024. Sample selection was based on titres from our patient archive. Samples in this archive were stored from 2000 onward and were obtained from patients every time they visited the outpatient clinic (most patients visited the outpatient clinic at least 3-4 times/year). Antigen-specificity of ANCA was determined as described before [9]. In 34 of the selected C-ANCA samples (*n* = 39), PR3-ANCA were detectable, while in 25 of the selected P-ANCA samples (*n* = 40) MPO-ANCA were detectable.

Next to these AAV sera, 5 distinct series of control samples were included. First, sera of healthy controls (*n* = 40) were included. Second, sera with antinuclear antibodies (ANA) were included to examine ANA interference. ANA patterns and titres were determined by routine ANA IIF analysis using Hep-2000 cells as a substrate (Immuno Concepts, Sacramento, CA, USA). These ANA controls consisted of sera with a homogenous ANA in three distinct titres (1/80, *n* = 6; 1/320, *n* = 7; 1/1280, *n* = 7), and sera with a speckled pattern (*n* = 5), an atypic speckled pattern (SSA-pattern; *n* = 4), a centromere pattern (*n* = 4), and a nucleolar pattern (*n* = 2). The nonhomogenous ANA sera all had a titre of 1/1280. Third, sera (*n* = 11) with antimitochondrial antibodies (AMA) were included to examine AMA interference. AMA were originally detected by routine IIF on liver/kidney/stomach slides (Scimedx, Denville, NJ, USA) in a 1/20 dilution and confirmed as reactive with E2-component of the pyruvate dehydrogenase complex (Euroimmun, Lübeck, Germany). Fourth, sera (*n* = 9) with a C-ANCA pattern (titres 1/16–1/1024) on ethanol-fixed ANCA slides (INOVA) due to reactivity with bactericidal permeability-increasing protein (BPI) as determined by ELISA (Euro Diagnostica, Lund, Sweden). Fifth, sera (*n* = 22) with an atypical ANCA staining pattern on ethanol-fixed ANCA slides (INOVA). The sera of the fourth and fifth control cohorts were all negative for PR3- and MPO-ANCA and came from patients that did not have AAV.

Since for the analyses on patient material sera were obtained for diagnostic purposes and the rest-serum was used in an anonymous way, ethical approval and informed consent was not necessary according to the Dutch guidelines. All sera were stored at −30°C until analysis.

### 2.2. ANCA Detection

ANCAs were detected in parallel on ethanol- and formalin-fixed neutrophils (Medipan, Berlin, Germany) by IIF. For both substrates, the sera were diluted 1/20; no further titration was performed. The assays were performed according to the manufacturer's instructions. Importantly, these assays were specifically designed for automatic reading in the AKLIDES system. In particular, the mounting medium contained DAPI to enable automatic focussing.

ANCA detection was first performed by AKLIDES. The system consists of a combination of a fluorescence microscope with an LED light source, a scan stage, a camera, and a personal computer containing the AKLIDES software (Medipan). The software, version AKLIDES 1.1 ANCA module: Build 47 (March 2011), automatically reads out images by controlling scan positions (*x*- and *y*-directions), focussing on the DAPI-staining (*z*-direction), calibration and recording of the fluorescence signal. For ANCA detection, 10 scan positions per well appeared optimal (data not shown). The AKLIDES software automatically analyses the intensity and structure of the fluorescence signal for each sample. Positive-negative differentiation, a nuclear-cytosolic localisation and assignment of ANCA fluorescence to C- or P-ANCA, or ANA-dots is provided. If a pattern could not be assigned to a positive sample, the score undetermined is given.

The reproducibility of the automatic pattern recognition was determined by the manufacturer (Medipan) by analysing one sample with PR3-ANCA and one sample with MPO-ANCA three times in three different dilutions on both ethanol- and formalin-fixed neutrophils. The PR3-ANCA revealed 8x a C-ANCA and 1x an undetermined pattern on ethanol-fixed neutrophils and 9x a C-ANCA on formalin-fixed neutrophils; the MPO-ANCA revealed 9x a P-ANCA and 9x a C-ANCA pattern on both substrates, respectively. Overall reproducibility is therefore >97%.

Second, ANCA fluorescence was judged by routine fluorescence microscopy using a Zeiss microscope with an LED light source (Zeiss, Oberkochen, Germany). All slides were evaluated by two observers blinded for the results obtained by routine analyses and AKLIDES. In case of a difference in opinion, a third observer was decisive.

## 3. Results

### 3.1. ANCA Pattern Recognition in Sera of Patients with AAV: Visual Scoring

**Figure 1:**
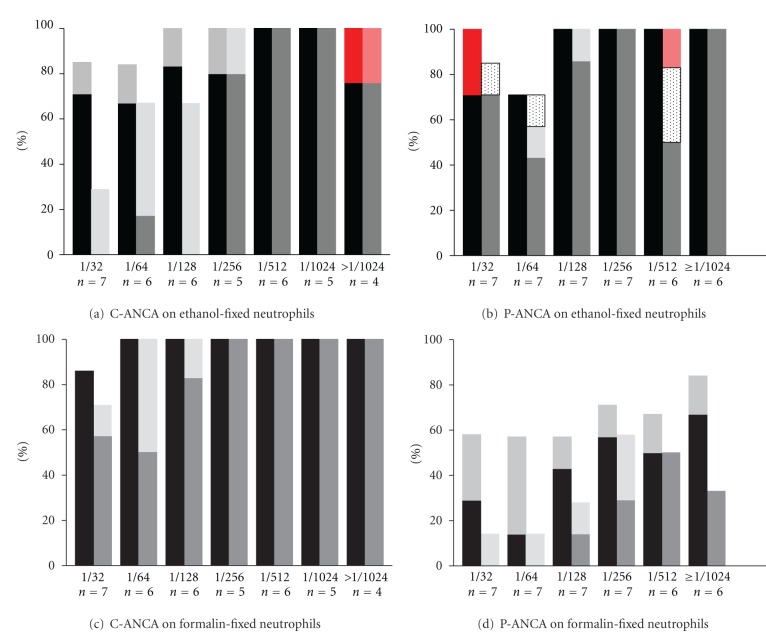
Pattern recognition by AKLIDES in sera of ANCA-associated vasculitis patients with C-ANCA/PR3-ANCA (*n* = 39; (a) and (c)) and P-ANCA/MPO-ANCA (*n* = 40; (b) and (d)) on ethanol-fixed neutrophils ((a) and (b)) and formalin-fixed neutrophils ((c) and (d)). Notably, P-ANCA samples are expected to reveal a C-ANCA pattern on formalin-fixed neutrophils (d). ANCA patterns were scored by routine fluorescence microscopy (black bars represent unequivocal, correct pattern recognition; intermediate grey bars on top of the black bars represent weak, but correct pattern recognition) and by AKLIDES (dark grey bars represent unequivocal, correct pattern recognition; light grey bars on top of the dark grey bars represent weak, but correct pattern recognition). Red and pink bars represent erroneous pattern recognition by routine fluorescence microscopy and AKLIDES, respectively, dotted bars indicate that the pattern was undetermined by AKLIDES. Data are expressed as percentage for each titre category; titres were historically measured during routine diagnostics.

**Figure 2:**
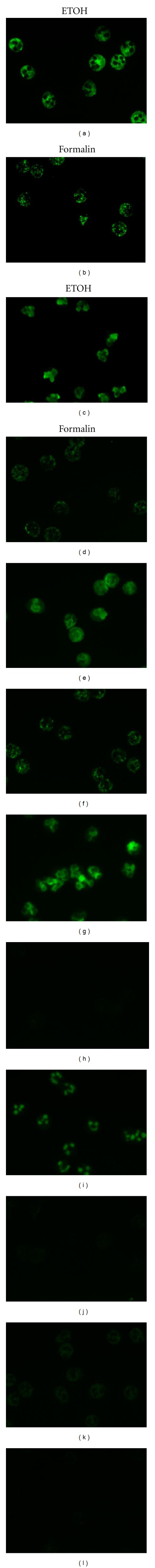
Images acquired by AKLIDES: typical C-ANCA on ethanol-fixed neutrophils (a) and formalin-fixed neutrophils (b); typical P-ANCA on ethanol-fixed neutrophils (c) and switch to C-ANCA on formalin-fixed neutrophils (d); expected C-ANCA revealing an atypical pattern by visual score on ethanol-fixed neutrophils and erroneously scored P-ANCA by AKLIDES (e), but consistent C-ANCA on formalin-fixed neutrophils (f); P-ANCA on ethanol-fixed neutrophils erroneously scored C-ANCA by AKLIDES (g) and consistent negative on formalin-fixed neutrophils (h); intermediate positive (1/320) homogenous ANA reported as P-ANCA on ethanol-fixed neutrophils by AKLIDES (i), being consistently negative on formalin-fixed neutrophils (j); BPI-ANCA revealing a weak C-ANCA pattern by visual score on ethanol-fixed neutrophils (k), scored negative by AKLIDES on both ethanol- and formalin-fixed neutrophils (l).

The results of ANCA pattern recognition, presented according to the titres measured by routine ANCA IIF, are summarized in Figure 1. Visual scoring of the Medipan slides with ethanol-fixed neutrophils revealed that in 36/39 samples (92.4%) the expected C-ANCA was observed (Figures 1(a) and 2(a)), while in 36/40 samples (90%) the expected P-ANCA was observed (Figures 1(b) and 2(c)). One C-ANCA sample (titre >1/1024) and two P-ANCA samples (titre 1/32) revealed an atypical pattern. Only samples with a low titre (1/32-1/64) were negative in the visual scoring of the Medipan slides. Altogether, the concordance in terms of pattern recognition with the historically performed routine ANCA IIF was 91.1%.

Visual scoring of the formalin-fixed neutrophils revealed in 38/39 of the samples (97.4%) the expected C-ANCA pattern (Figures 1(c) and 2(b)); the missed sample (titre 1/32) scored negative. The expected switch from P-ANCA to C-ANCA on formalin-fixed neutrophils, however, was observed in only 25/40 samples (62.5%); this was not related with the titres measured by historically performed routine ANCA IIF (Figures 1(d) and 2(d)). The samples that did not show a C-ANCA pattern on formalin-fixed slides (*n* = 14) were predominantly MPO-ANCA-negative (64.3%), while the majority of C-ANCA-positive samples (*n* = 26) were MPO-ANCA-positive (76.9%) as detected by an antigen-specific assay.

### 3.2. ANCA Pattern Recognition by AKLIDES in Sera of Patients with AAV

The AKLIDES system scored a C-ANCA on ethanol-fixed neutrophils in 29/39 samples (74.4%). Negative results were restricted to the samples with low/median titres (1/32–1/128). The positive samples in these titre categories revealed only a weak intensity (Figure 1(a)). Only one apparent mismatch (P-ANCA) was observed in a sample with a high titre (>1/1024); this sample was visually also scored in the wrong pattern, that is, atypical ANCA (Figure 2(e)). The C-ANCA was better recognized by the AKLIDES system on the formalin-fixed slides: 37/39 samples (94.9%) were positive (Figure 1(c)). Again, weak-positive results were limited to samples with low/median titres.

A P-ANCA on ethanol-fixed neutrophils was scored by the AKLIDES system in 32/40 samples (80.0%); four samples were scored as undetermined and one sample (titre 1/512) was erroneously scored C-ANCA (Figures 1(b) and 2(g)). The three negative samples had low titres (1/32-1/64) on ethanol-fixed neutrophils. As compared to the visual scoring, the expected P-ANCA to C-ANCA switch on formalin-fixed neutrophils was not very well recognized by the AKLIDES system (Figure 1(d)). Only 13/40 samples (32.5%) revealed a P-ANCA to C-ANCA switch. All other samples were negative.

The concordance in pattern recognition on ethanol-fixed neutrophils between the AKLIDES system and the visual scoring was 79.7%. The discordant results were mainly due to the lower sensitivity of the AKLIDES system for the low titre C-ANCA samples (*n* = 7; Figure 1(a)) and the P-ANCA samples that were scored as undetermined (*n* = 4; Figure 1(b)). As mentioned, two apparent pattern mismatches were reported by the AKLIDES system (Figures 1(a), 1(b), 2(e), and 2(g).

### 3.3. ANCA Pattern Recognition in Sera of Healthy Controls

Visual scoring of the slides with ethanol-fixed neutrophils revealed negative results in 35/40 samples (87.5%). A P-ANCA (*n* = 1) and weak C-ANCA pattern (*n* = 4) was recognized in the positive samples. The AKLIDES system revealed negative results in 38/40 samples (95%). One sample scored as weak C-ANCA and another sample as undetermined. The concordance was 85%. All samples were reported negative on the formalin-fixed neutrophils by both the visual scoring and the AKLIDES system.

### 3.4. Interference of ANCA Pattern Recognition in Sera Containing ANA or AMA

ANA, and to a lesser extent AMA, are known to hamper ANCA pattern recognition on ethanol-fixed neutrophils. In particular, homogenous ANA may obscure the P-ANCA pattern. As summarized in Table 1, low-titre homogenous ANA could visually be recognized as such, but high-titre homogenous ANA may appear as P-ANCA when analysed in a low serum dilution. The AKLIDES system was not able to discriminate between a nuclear staining pattern and a P-ANCA pattern, independent of the ANA titre (Figure 2(i)). This resulted in an overall concordance of only 40%. Importantly, the majority of these samples were negative on formalin-fixed neutrophils (Figure 2(j)). Only 1 and 2 sample(s) gave a C-ANCA in the visual score and the AKLIDES system, respectively, (data not shown). Both samples had a 1/1280 homogenous ANA that was reported as P-ANCA by both the visual score and the AKLIDES system.

As can be concluded from Table 1, also samples with most other ANA patterns or AMA hampered ANCA pattern recognition on ethanol-fixed neutrophils, either visually (76.9%) or by the AKLIDES system (65.4%). Overall concordance in these samples was 57.7%. On formalin-fixed neutrophils the visual score revealed only a single C-ANCA in a sample with a 1/1280 speckled ANA, while the AKLIDES system reported a C-ANCA in 3 samples with AMA; two of these were also reported as P-ANCA on ethanol-fixed neutrophils. All other samples were negative on formalin-fixed neutrophils (data not shown).

### 3.5. ANCA Pattern Recognition in Sera Containing BPI-ANCA or Atypical ANCA

Sera with BPI-ANCA consistently revealed a C-ANCA in the visual score on ethanol-fixed neutrophils (Table 2 and Figure 2(k)); two of these samples also were weakly C-ANCA-positive on formalin-fixed neutrophils. Interestingly, the AKLIDES system reported negative results (*n* = 6) or ANA-dots (*n* = 3), but not C-ANCA on ethanol-fixed neutrophils (Table 2); again, two samples were weakly C-ANCA-positive on formalin-fixed neutrophils.

An atypical ANCA, as recognized in 22 samples by IIF on INOVA slides, was visually only found in 5 samples on Medipan slides containing ethanol-fixed neutrophils. The other samples were negative (*n* = 8) or revealed a C-ANCA (*n* = 9). Two samples with a C-ANCA also were weakly C-ANCA positive on the formalin-fixed neutrophils (data not shown). The AKLIDES system does not report atypical ANCA. Interestingly, the majority of these samples were scored negative (*n* = 18), while 4 samples were scored as P-ANCA (*n* = 1), C-ANCA (*n* = 2), or as undetermined (*n* = 1) (Table 2). All samples were reported negative by the AKLIDES system on formalin-fixed neutrophils (data not shown).

## 4. Discussion

In this study, we have evaluated the first automated system for ANCA pattern recognition on slides with ethanol- and formalin-fixed neutrophils. The data obtained in PR3-ANCA sera of AAV patients reveal that the AKLIDES system lacks sufficient sensitivity to ethanol-fixed neutrophils, but these sera are very well recognized as C-ANCA on formalin-fixed neutrophils. MPO-ANCA sera of AAV patients, however, are best recognized as P-ANCA on ethanol-fixed neutrophils, while they are poorly recognized as C-ANCA on formalin-fixed neutrophils. In control sera the most apparent interference was observed in sera with a homogenous ANA. In contrast to visual scoring, the AKLIDES system could not differentiate between P-ANCA and a nuclear pattern in most of these samples. On the other hand, the AKLIDES system can discriminate C-ANCA due to reactivity to PR3 (C-ANCA) or to BPI (negative), while this appeared visually impossible.

In the current study sample selection and interpretation of results were based on historically obtained data in routine diagnostics. This approach has two caveats. First, ANCA detection was historically performed on ethanol-fixed neutrophil slides of INOVA, while the AKLIDES system is restricted to Medipan slides. Differences between the historically obtained routine results and the current visual score, therefore, can be attributed to the use of distinct substrates. The reduced sensitivity (~95%) of the Medipan slides for both C- and P-ANCA may, however, also be due to quenching of the fluorescent signal because the slides were first analysed by the AKLIDES system and next, visually by routine fluorescence microscopy. Second, selection of P-ANCA samples was based on MPO-reactivity at the time of diagnosis. The samples included, however, were P-ANCA-positive, but not necessarily MPO-ANCA-positive. This seems to be the explanation for many samples not giving a C-ANCA pattern on formalin-fixed neutrophils. The observed P-ANCA is most likely due to antibody reactivity to other minor granular components of neutrophils [10].

According to the international consensus on ANCA detection for AAV, the first step is to be performed by IIF [5]. As such, the IIF is primarily performed as a screening assay to select samples that require reflex testing for MPO- and PR3-ANCA. For this purpose, the sensitivity should be high, while the immunofluorescence pattern is irrelevant. Our current study shows that when positivity is defined as any reactivity on ethanol-fixed slides and/or formalin-fixed slides, the sensitivity for AAV of the AKLIDES system is 94%, while the sensitivity of the visual scoring is 96%. It should be stressed, however, that the samples included were not diagnostic samples and were not randomly selected. Therefore, this study is not appropriate to calculate either sensitivity or specificity for AAV, but at least reveals that the technical sensitivity of automatic reading equals visual scoring. A small study recently published revealed an overall agreement in positive and negative results between visual expert reading and automated AKLIDES interpretation of 87% [11]. Unfortunately, this study lacked any clinical information on the 46 samples included.

Like in ANA detection [12], the immunofluorescence pattern of ANCA has predictive value for the antigen specificity of the ANCA [13]. The international consensus prescribes that, whatever the ANCA pattern, testing for both MPO- and PR3-ANCA should be performed [5]. However, the specific combination of a C-ANCA/PR3-ANCA or P-ANCA/MPO-ANCA increases the clinical utility of ANCA diagnostics [6]. Therefore, correct pattern recognition is important and for this the reactivity on ethanol-fixed slides is leading. In this respect, it can be concluded from our study that the automatic recognition of the C-ANCA pattern on ethanol-fixed slides lacks sufficient sensitivity. This is accompanied by the possibility to discriminate the IIF pattern of BPI-ANCA from PR3-ANCA by the AKLIDES. For ANCA diagnostics, however, the first issue is more important than the latter. In a pilot study, recognition of the C-ANCA pattern on ethanol-fixed slides could be increased from 29/39 samples (74.4%) to 35/39 samples (89.7%). At the cost of the distinction between BPI- and PR3-ANCA, this software modification did not influence the reactivity in the healthy control cohort or the P-ANCA reactivity in the respective AAV cohort (data not shown). Apparently, changes in the software of the AKLIDES system enable to improve correct pattern recognition.

According to the international consensus, titration of ANCA IIF is optional [5]. Titration is recommended if serum samples are positive by IIF but negative for MPO- or PR3-ANCA, for followup of such patients, and to distinguish ANCA from interfering ANA. The latter might be the cause of the many P-ANCA reports by AKLIDES in samples with a homogeneous ANA. Titration of the samples might have enabled a better distinction of the nuclear staining pattern. The desire to quantitate the ANCA by titration can possibly be replaced by measuring fluorescence intensity. It has been reported that the higher the ANCA titre as measured by IIF, the higher the likelihood for having AAV [14]. It remains to be determined whether this also holds for fluorescence intensity as determined by the AKLIDES system. The clinical utility of ANCA quantification during followup is still a matter of discussion [1, 15, 16]. In the early days of image analysis, this technique performed slightly better in predicting relapses than routine IIF [17]. Data obtained by the novel systems are lacking.

## 5. Conclusion

Altogether it can be concluded that automatic reading of IIF by pattern recognition software has paved the way for a new discussion on the role of IIF in autoimmune diagnostics. The argument that IIF is hampered by subjective interpretation and poor interlaboratory reproducibility seems to be outdated. Although our data reveal that ANCA pattern recognition requires further improvements, the current achievements in combination with the possibility to adapt the software are very promising for the near future. 

## Figures and Tables

**Table 1:** Interference of ANCA pattern recognition in sera containing ANA or AMA.

	Visual score	AKLIDES	Concordance
ANA homogenous			
1/80 (*n* = 6)	3 negative, 3 nuclear	3 negative, 3 P-ANCA	3/6 (50.0%)
1/320 (*n* = 7)	2 negative, 5 nuclear	2 negative, 5 P-ANCA	2/7 (28.6%)
1/1280 (*n* = 7)	4 nuclear, 3 P-ANCA	7 P-ANCA	3/7 (42.9%)

ANA speckled (*n* = 5)	2 negative, 2 nuclear, 1 atypic	2 negative, 2 P-ANCA, 1 ANA-dots	3/5 (60.0%)
Atypical speckled (*n* = 4)	1 negative, 3 P-ANCA	1 negative, 2 P-ANCA, 1 undetermined	3/4 (75.0%)
Centromere (*n* = 4)	3 nuclear, 1 atypic	4 ANA-dots	3/4 (75.0%)
Nucleolar (*n* = 2)	2 negative	2 negative	2/2 (100%)

AMA (*n* = 11)	1 negative, 2 P-ANCA, 2 C-ANCA, 4 atypic, 2 nuclear	4 negative, 3 P-ANCA, 1 C-ANCA, 3 ANA-dots	4/11 (36.4%)

AMA: antimitochondrial antibodies; ANA: antinuclear antibodies; ANCA: antineutrophil cytoplasmic antibodies.

NB: a nuclear pattern in the visual score is considered concordant with ANA-dots by the AKLIDES system.

**Table 2:** ANCA pattern recognition in sera containing BPI-ANCA or atypical ANCA.

	Visual score	AKLIDES	Concordance
BPI-ANCA (*n* = 9)	9 C-ANCA	6 negative, 3 ANA-dots	0/9 (0.0%)
Atypical ANCA (*n* = 22)	8 negative, 9 C-ANCA, 5 atypical ANCA	18 negative, 2 C-ANCA, 1 P-ANCA, 1 undetermined	10/22 (45.5%)

ANA: antinuclear antibodies; ANCA: antineutrophil cytoplasmic antibodies; BPI: bactericidal permeability-increasing protein.
